# Fishing Out a Bone From the Neck

**DOI:** 10.7759/cureus.58010

**Published:** 2024-04-10

**Authors:** Trinyanasuntari Munusamy, Seenu Uthaya Kumar, Komathi Ramachandran, Mazita Ami

**Affiliations:** 1 Otolaryngology - Head and Neck Surgery, Graduate School of Medicine, KPJ University, Nilai, MYS; 2 Otolaryngology - Head and Neck Surgery, Hospital Putrajaya, Putrajaya, MYS; 3 Otolaryngology - Head and Neck Surgery, KPJ Klang Specialist Hospital, Klang, MYS

**Keywords:** stingray, food bolus, ct neck, esophageal perforation, fish bone

## Abstract

Foreign body ingestion is a common medical issue in Asian populations. Fish bones are the most commonly ingested foreign bodies due to the practice of cooking fish whole with bones intact, unlike in Western countries where fish are typically prepared as fillets or patties. Patients who have swallowed fish bones usually present with foreign body sensations, odynophagia, and pricking sensations during deglutination. Fish bones can generally be removed in an outpatient setting, but in some cases, patients must be placed under general anesthesia, where rigid esophagoscopy is performed. In some cases, neck exploration is required to extricate the bone. Here, we report the case of a 71-year-old man who underwent neck exploration for a 2.1 cm fish bone lateral to his thyroid cartilage, penetrating the left thyroid lobe.

## Introduction

Fish bones are the most commonly ingested foreign bodies in Asian patients [1-4]. Numerous cases have already been published describing ingested foreign bodies in the upper aerodigestive tract. However, only a small number of those foreign bodies had perforated the esophagus and migrated extraluminally. Such a migration can cause disastrous complications and warrants further investigation [1].

## Case presentation

**Figure 1:**
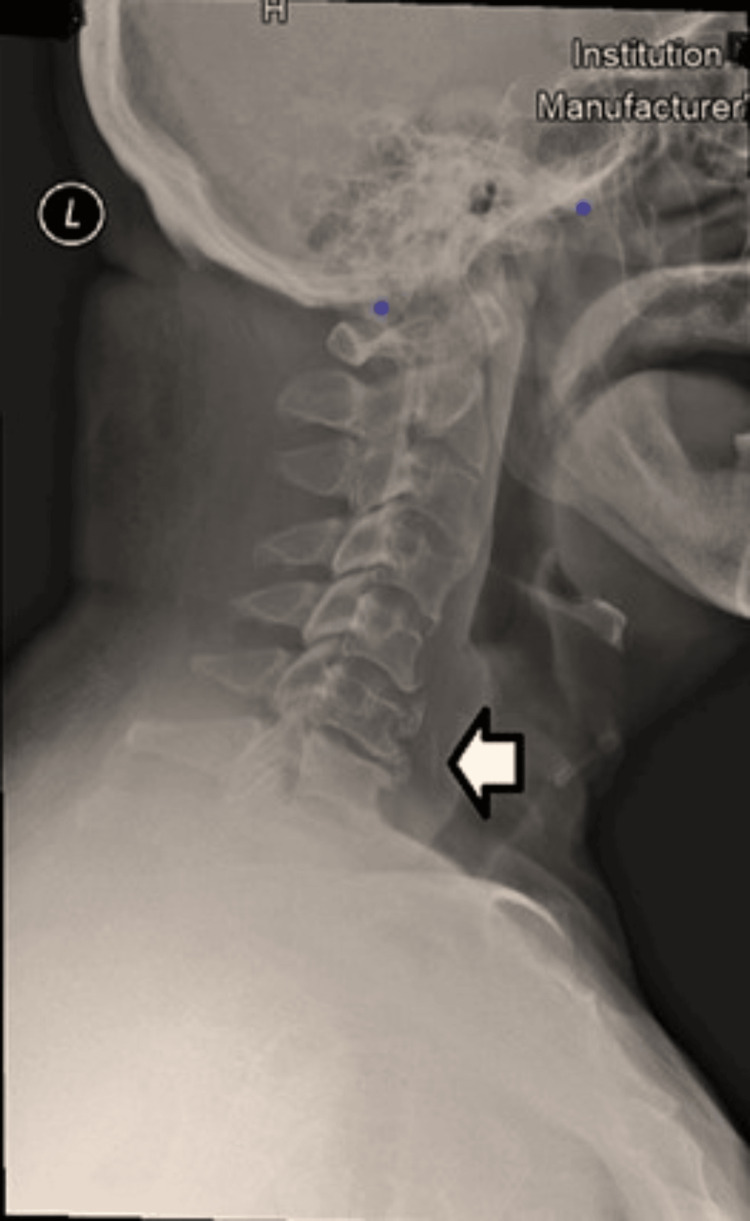
Plain lateral neck X-ray showing the fish bone at the level of the C6 vertebral body.

A 71-year-old Malay gentleman with multiple comorbidities including diabetes mellitus and ischemic heart disease on dual antiplatelet was referred to the otorhinolaryngology department with complaints of alleged fish bone (stingray) ingestion four days ago. The patient had failed to manually remove the fish bone by swallowing a bolus of rice. He presented with odynophagia, pricking pain more upon swallowing over the left side of the throat, and hoarseness. On examination, he was not in respiratory distress and vital signs were stable. Physical examination of the neck revealed warm and mild tenderness over the upper one-third of the left lateral cervical region extending to the postauricular region. Upon flexible nasopharyngeal laryngeal scope and flexible transnasal esophagoscopy, no foreign body was seen. A pooling of saliva was noted at the right pyriform fossa. Lateral soft tissue neck radiography noted a linear radiopaque foreign body at the C6 level (Figure 1).

**Figure 2:**
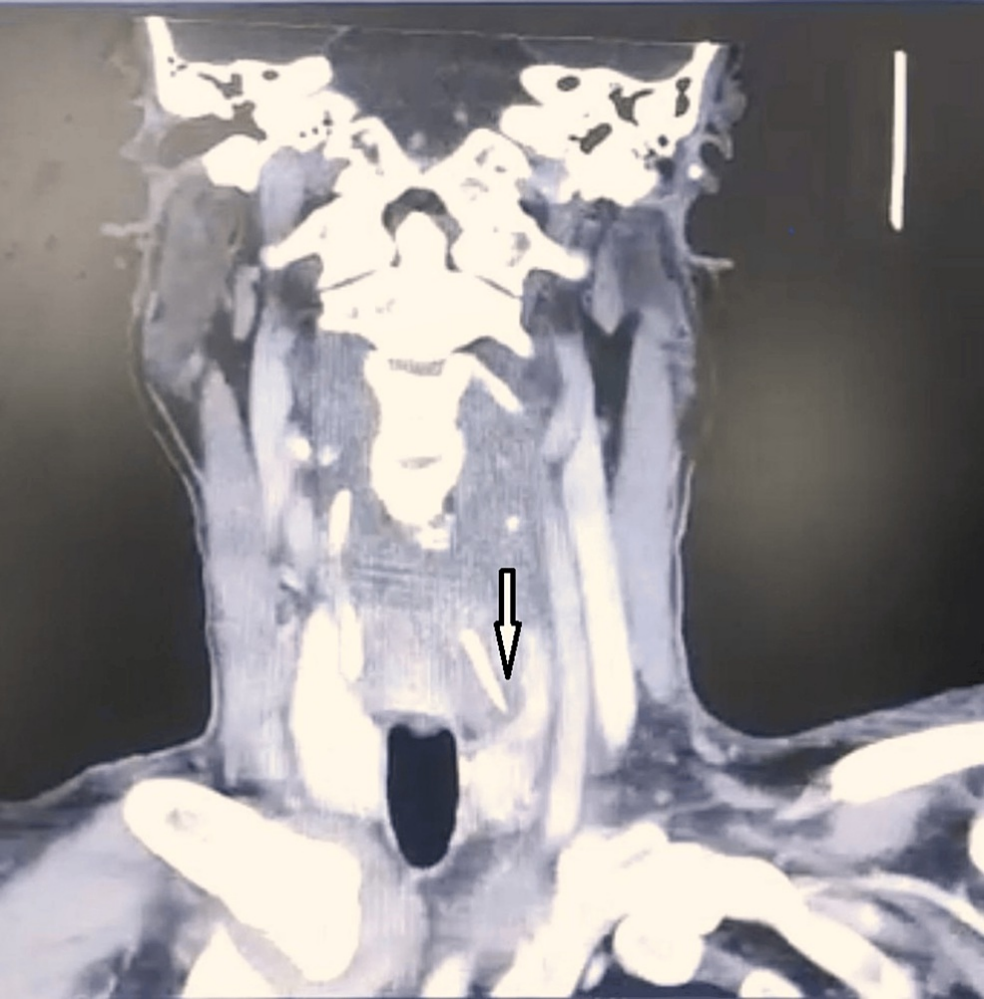
CT of the neck (coronal cut contrast-enhanced) showing the location of the fish bone over the left superior horn of the thyroid cartilage.

Unfortunately, the patient was also diagnosed with COVID-19 category 2A during the hospitalization; hence, conservative management with intravenous rocephin and metronidazole was commenced. A CT of the neck with contrast noted left supraglottic laryngeal wall collection and two suspicious hyperdense foreign bodies at the left retrocricoid and left superior horn of thyroid cartilage not involving the esophagus (Figure 2).

**Figure 3:**
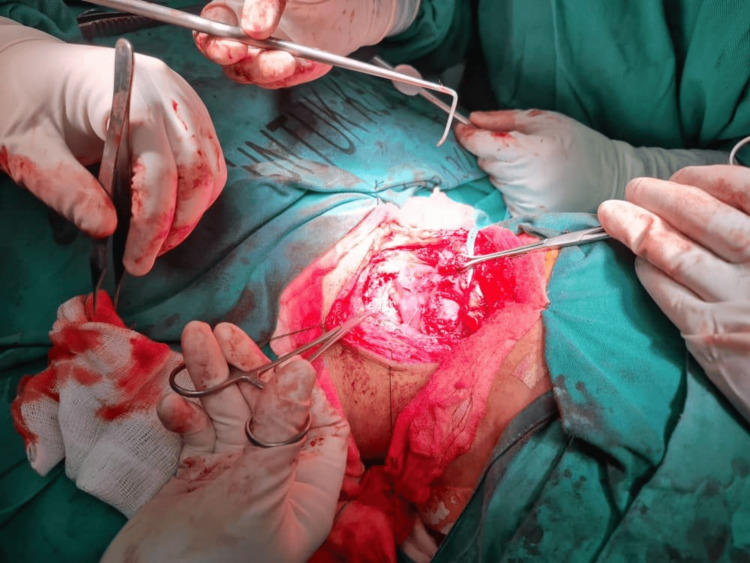
Neck dissection showing the fish bone found lateral to the thyroid cartilage.

**Figure 4:**
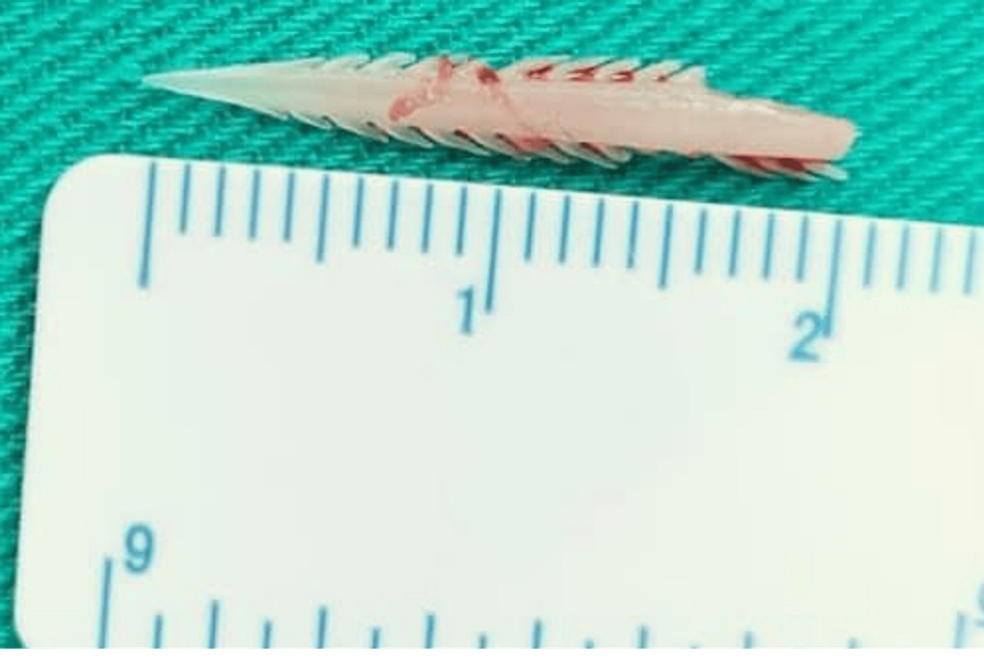
The 2.1 cm serrated fish bone discovered over the lateral thyroid cartilage, penetrating the left thyroid lobe.

It was decided to proceed with surgery. Under general anesthesia, neck exploration was done using a direct laryngoscope and esophagoscope. The direct laryngoscope revealed normal findings. The esophagoscope noted inflamed mucosa over the posterior wall of the esophagus at the cricopharyngeal level, 15 cm from the upper Incisor. Following neck exploration, a left lateral utility neck incision was made, running from the left posterior border of the sternocleidomastoid to the upper border of the thyroid cartilage at the midline, along the skin crease. A subplatysmal flap was raised superiorly until the lower border of the mandible, inferiorly until the upper border of the clavicle, anteriorly until midline, and laterally until the posterior border of the left sternocleidomastoid. Neck dissection was done until reaching the thyroid lobe (Figure 3). A serrated fish bone measuring 2.1 cm was found lateral to the thyroid cartilage, penetrating the left thyroid lobe, which was edematous, and yellowish fluid was noted in the left thyroid lobe (Figure 4).

The left recurrent laryngeal nerve was identified and preserved. A Radivac drain size 12 was inserted and anchored to the skin. Post-surgery, the patient was kept nil per oral and started Ryle’s tube feeding till a gastrograffin study was scheduled one week later. This was due to the esophagoscope finding of inflamed mucosa over the posterior esophagus. The gastrograffin study showed no significant abnormality in the fluoroscopic study and a patent upper esophagus. The patient returned to his normal diet and was discharged well on day eight post-operation. The drain was removed on day five post-surgery as 30-40 cc of hemoserous fluid was drained throughout. The patient developed transient thyroiditis post-surgery with a free T4 of 19.8 µg/dL and thyroid-stimulating hormone (TSH) of 0.036 mIU/L. This was due to thyroid gland manipulation intraoperatively. He was subsequently reviewed in the outpatient clinic after two weeks. He appeared well with no symptoms and normal thyroid function, with free T4 of 7.9 µg/dL and TSH of 0.34 mIU/L. He was subsequently discharged from follow-up.

## Discussion

Otorhinolaryngological cases of fish bone ingestion are most often encountered in countries with high rates of fish consumption, especially in coastal countries in Asia and the Mediterranean [1,3,5]. Data indicate that fish bones account for most upper aerodigestive tract foreign bodies (50%-90% of total foreign bodies) among patients living in Asian countries [3,5]. Fish bone ingestion is most prevalent in children, especially those aged two to four years, and middle-aged adults. In adult cases, ingestion of fish bones occurs more commonly after age 40 or older because of deterioration of the swallowing function [5]. Besides age, other factors that contribute to swallowing deterioration include pre-existing pathologies of strictures (37%), malignancy (10%), esophageal rings (10%), and achalasia (2%) [5,6]. However, in some cases, no pathological predisposition is present. Cases of ingested foreign bodies are also reported more frequently in patients of advanced age, patients with intellectual disabilities or psychiatric disorders, and patients who wear dentures.

Patients who have ingested fish bones generally present within 24 hours to the emergency department and are referred to the otorhinolaryngology department. As in our case, many patients practice improper measures after feeling a retained foreign body in their throat, such as swallowing rice balls, leeks, and steamed bread. This patient forcibly swallowed rice balls following ingestion of the fish bone, leading to the migration of the foreign body and subsequent additional complications. Fish bones are commonly found impacted at the base of the tongue or in the palatine tonsils or vallecula, where they can be easily removed in an outpatient procedure. Other sites, such as the pyriform fossa, epiglottis, and esophagus (5% combined incidence), can also be involved [2,3,5].

Diagnosing fish bone ingestion involves taking a detailed patient history and imaging via lateral neck X-rays, neck CT scans, or flexible and rigid endoscopy. A neck X-ray with a soft tissue view (lateral and anteroposterior) helps locate radio-opaque foreign bodies such as fish bones. The sensitivity and specificity of X-rays for detecting fish bones have been reported as 39% and 72%, respectively [2-4]. In our case, a neck CT was performed to assess the bone’s location, size, and orientation, as well as its relationship to other vital structures in the neck. CT scans are also useful in cases with complications related to fish bone impaction or negative investigation modalities with positive symptoms [4]. CT scans are preferred in such cases because the resulting images can serve as a map during surgical exploration. According to Weber et al., CT scans have 100% sensitivity and 91% specificity, indicating their superior ability for the diagnostic evaluation of ingested and migrated foreign bodies [6].

According to Thomas et al., the process by which foreign bodies are propelled through neck tissues of the neck is not known, but one proposed cause is a sequence of esophageal peristalsis and neck movements combined with carotid pulsations [1]. Tissue reaction to a foreign body, abscess, and infection could likewise have an impact on extraluminal propulsion. Shroff et al. attributed fish bone migration to several factors, including orientation, bone shape, and cricopharyngeal muscle contraction, concluding that a horizontally oriented fishbone is more likely to migrate extraluminally [2]. It is unknown whether the practice of swallowing rice, olive oil, or bread to push the foreign body into the stomach increases the likelihood of extraluminal migration of the foreign body [1].

Swallowed fish bones tend to migrate to the thyroid gland, carotid artery, mediastinum, or subcutaneous tissue because of the constant contraction and relaxation of the pharyngeal musculature, esophageal peristalsis, and surrounding tissue reactions [1,3]. Resulting complications can include deep neck abscesses, vascular complications (including vascular esophageal fistula), puncture of the carotid artery, thromboembolism, and thyroid gland retention. Very rarely, fish bones tracked down to the gastrointestinal tract result in pseudotumor of the liver [2].

Complications may arise depending on the direction and site of the migrating fish bone. In this case, the 2.1 cm fish bone was found lateral to the thyroid cartilage, penetrating the left thyroid lobe, and it appeared to be edematous. This patient had transient episodes of hyperthyroidism due to the insult to the left thyroid lobe. According to Thomas et al., the shape of the foreign body is the most important factor in the pathology of migration. Saw-toothed fish bones are capable of penetrating deep into the retropharyngeal space. Fortunately for this patient, there were no extensive abscess formations or involvement of the deep spaces of the neck [1].

## Conclusions

Good clinical judgment is required when diagnosing a migrating foreign body. To prevent life-threatening consequences, early intervention should be done if the foreign body is not located endoscopically and extraluminal migration is suspected. A CT scan is the best diagnostic tool to preoperatively determine the exact nature and position of a swallowed fish bone in a case where complications are anticipated. Our goal in writing this case report is to increase knowledge about this illness.
